# Cost Analysis of Selected Patient Categories within a Dermatology Department Using an ABC Approach

**DOI:** 10.5539/gjhs.v8n6p234

**Published:** 2015-11-17

**Authors:** Šárka Papadaki, Boris Popesko

**Affiliations:** 1 Faculty of Management and Economics, Tomas Bata University in Zlin, Mostni 5139, Zlin, 760 01, Czech Republic

**Keywords:** hospital, costing, ABC method, modern costing methods, M10, M21

## Abstract

**Background:**

Present trends in hospital management are facilitating the utilization of more accurate costing methods, which potentially results in superior cost-related information and improved managerial decision-making. However, the Activity-Based Costing method (ABC), which was designed for cost allocation purposes in the 1980s, is not widely used by healthcare organizations. This study analyzes costs related to selected categories of patients, those suffering from psoriasis, varicose ulcers, eczema and other conditions, within a dermatology department at a Czech regional hospital.

**Methods:**

The study was conducted in a hospital department where both inpatient and outpatient care are offered. Firstly, the diseases treated at the department were identified. Further costs were determined for each activity using ABC. The study utilized data from managerial and financial accounting, as well as data obtained through interviews with departmental staff. Using a defined cost-allocation procedure makes it possible to determine the cost of an individual patient with a given disease more accurately than via traditional costing procedures.

**Results:**

The cost analysis focused on the differences between the costs related to individual patients within the selected diagnoses, variations between inpatient and outpatient treatments and the costs of activities performed by the dermatology department. Furthermore, comparing the costs identified through this approach and the revenue stemming from the health insurance system is an option.

**Conclusions:**

Activity-Based Costing is more accurate and relevant than the traditional costing method. The outputs of ABC provide an abundance of additional information for managers. The benefits of this research lie in its practically-tested outputs, resulting from calculating the costs of hospitalization, which could prove invaluable to persons involved in hospital management and decision-making. The study also defines the managerial implications of the performed cost analysis for the hospital management. Based on the analysis results, it is possible to standardize activities and performance appraisal (Benchmarking), and provide all necessary information for hospital budgeting practices, especially Activity-Based Budgeting (ABB).

## 1. Introduction

Hospitals worldwide struggle with the combination of limited resources, increasing costs and greater demand for services. Studies show evidence of a rise in healthcare costs in recent decades (Chernew et al., 2003; Caughey & Burchfield, 2014). Higher spending on healthcare also negatively affects the economic measures taken by healthcare organizations. Hospitals and other healthcare organizations are, due to the negative trend in costs, under pressure to more effectively manage activities and outputs performed with limited resources.

Many studies published in the past few decades have highlighted the ever greater importance of effective cost control and the necessity to apply advanced costing methods for more accurate analysis of the costs of providing services and handling patients within hospital organizations. Cao et al. (2006) state that in order to realize effective cost control, a practical and accurate cost accounting system is indispensable to hospitals. Edbrooke et al. (1997) point out the increasing need for a method to obtain accurate patient costs. Ridderstolpe et al. (2002) state that a valid basis for calculation is increasingly necessary to control the cost of health care. Koyama (2000) states that the importance of obtaining accurate estimates of costs for medical services is increasingly recognized by hospital managers. Chapko et al. (2009) write that accurately estimating the costs of specific healthcare services and care for individual patients is critical to the efficient administration of healthcare systems, so as to prevent inappropriate payment incentives and to conduct research on health services. According to Rudawska (2009) hospitals achieved better competitive advantage if they properly manage their costs.

The literature documents various methods used to estimate costs for patient-related services provided by hospitals. These include the use of average costs obtained from dividing total annual expenditure by patient throughput (Bams & Miranda, 1985), the use of severity of illness and workload scoring systems (Zimmermann et al., 1993) and the use of billing systems (Finkler, 1982). Carvalho, Jericó and Castilho (2010) state that most hospitals applying cost management systems use the absorption method. This is the most common costing technique utilized across various industries (Popesko & Novák, 2011). The advantage of absorption costing is its broad applicability and ease of use. Nevertheless, absorption costing is often criticized for inaccuracy. Cao et al. (2006) comment that although the VBC (volume-based costing) method benefits from simplicity, it often returns approximate and inaccurate results.

The objective of the study presented here was to perform a cost analysis of a selected sample of patients with different diagnoses by utilizing an advanced Activity-Based Costing system. It has already been proven that standard, simple costing methods, such as division or absorption costing, often provide inaccurate results in estimating the cost of individual patients of DRGs (diagnosis-related groups). Applying the more sophisticated Activity-Based Costing method (ABC) could improve the accuracy of the estimated costs. The additional information provided by ABC also benefits decision-makers and managers. This paper presents the manner of application of the ABC approach within the dermatology department of a Czech regional hospital. It also compares the costs of the selected sample of patients within the chosen diagnoses, as calculated by ABC, with the insurance payments received by the institution and the costs determined by the traditional costing methods currently used at the hospital.

### 1.1 Literature Review

Approaches to estimating costs have been broadly divided into two categories: top-down and bottom-up (Carey & Burgess, 2000; Negrini et al., 2004). Top-down approaches use relative value units (RVU), hospitalization days, or another parameter to assign the total costs for a healthcare system to individual services. Bottom-up approaches, such as Activity-Based Costing (ABC), assess the amount of each resource that is used to produce an individual healthcare service, assigning costs accordingly to generate aggregate costs for a healthcare system (Chapko et al., 2009).

Problems related to the use of traditional costing methods, which caused distortions in indirect costs, and accounting reports, had been excessively discussed in literature. These methods normally do not provide managerial interpretation and definition of tasks determining the control of deviations related to specific problems. Strong criticism of the traditional costing concept was voiced by Kaplan and Johnson (1987). Even so, traditional costing methods are still frequently used in practice, mostly because of their simplicity and minimal data input requirements. The limitations of such traditional costing methods could prove more crucial when applying costing systems in service organizations. Rotch (1990) defined several reasons why cost allocation and implementing a costing system in a service organization might turn out to be more complicated.

Growing dissatisfaction with standard costing systems resulted in the creation of the Activity-Based Costing method, which was first introduced by Kaplan and Johnson (1987). ABC, widely recognized since the 1980s, improves the accuracy of cost allocation and the ability to measure the output of organizational activities. The most important difference between traditional costing and Activity-Based Costing systems is that overhead costs are not allocated using the surrogate allocation base, which often leads to arbitrary allocation (Drury, 2001). Instead, overhead cost allocation is made via the activities performed within the organization. Generally, ABC consists of two stages: costs are allocated to activities, and then activities are allocated to a product or service (Baker, 1998).

One of the earliest studies focusing on applications of ABC in a hospital was published by Helmi and Tanju (1991). The study was carried out in 1991, where the costs of a nursing station for three different patient types were calculated. Ramsey (1994) was responsible for the notion that ABC is not only an accounting system but also a strategic management tool. At present, many studies are being conducted on applying this method to the hospital environment. For example, Lievens (2003) describes the development and application of an ABC system for a radiotherapy department. Goldberg (2011) details the methodology implemented at a department of gastroenterology and hepatology. Aqyer (2007) describes the application of ABC at a urology department and suggests that the model could be introduced in other hospital departments.

Many authors describe the differences between traditional cost accounting and ABC accounting (Lin, 2007; Cao, 2006; Andrade, 1999). The greatest of these is that overhead costs are not allocated using the surrogate allocation base, which often leads to arbitrary allocation (Drury, 2001), but are allocated via the activities performed within the given organization. Generally, Activity-Based Costing is based on the concept that the performance of a service consumes activities that in turn consume resources (Goldberg, 2011). According to Wodchis (1999), traditional costing techniques allocate costs to all units based on an average unit cost, or pool indirect costs and allocate them to the various services in proportion to the volume of services or direct costs (Gujral, 2010). Andrade (1999) thinks ABC reflects the costs of operations in a company in a more accurate and consistent manner than traditional methods, and hence it has been increasingly implemented as an alternative to traditional costing. Agyar (2007) writes that ABC provides better quality information for management than traditional accounting. The biggest disadvantage presented in many studies is that traditional methods of cost accounting could overestimate or underestimate the costs of services, because overhead costs could vary with the complexity of delivering such services and not with the volume of services. (Hankins, 2004; Lucey, 2002; Aird, 1996). In order to more accurately determine the efficiency of the ABC model, a traditional cost accounting system has to be utilized in parallel (Agyar, 2007).

The general methodology for ABC implementation has been variously described. For example, Drury (2001) identifies four steps: (1) identifying the major activities taking place in an organization; (2) assigning costs to cost pools/cost centers for each activity; (3) determining the cost driver for each activity; (4) assigning the costs of activities to products according to their individual demands on activities. Gujral (2010) applied this method at a hospital in a hematopathology laboratory and described the procedure in the following 4 steps: (1) a cost that can be directly allocated to each test separately is identified. These are considered direct costs for each test. The annual direct cost for each test is determined; (2) costs that cannot be allied with a particular test but are related to all the tests in the laboratory are pooled together as common laboratory costs (indirect costs); (3) indirect costs are allocated to each test in proportion to the number of samples processed for each test. The annual indirect costs are determined; (4) direct costs and the proportionate share of indirect costs are added together and divided by the number of samples for that particular test. The sample cost of each test is thus determined.

ABC is related to the following terms - cost driver and cost object - and these basic concepts are discussed in many studies. According to Lievens (2003), cost drivers are used to allocate indirect costs to a product. Cao (2006) discusses the complexity of finding individual cost drivers that are essential to this method. Agyar (2007) presents two stages of cost drivers, a first and second order cost driver contrast, whereas Lievens (2003) uses three instead of two levels of cost drivers.

It is uncertain why ABC has not been more commonly adopted by hospitals worldwide when the benefits of its adoption are so widely accepted by experts. Despite the benefits of ABC application, actual usage by healthcare organizations is scarce. (Gujral, 2010) But still some studies are presented, for instance Selina (2013) presents application of ABC in Radiography center or Zhao (2013) discus the differences between traditional cost method and ABC.

According to Stouthuysen et al. (2009), implementing ABC is hampered by several serious limitations, such as the high complexity of the system and creating conditions for gathering non-financial information. They see the need for some degree of caution in advocating the use of ABC in hospitals. Lievens et al. (2003) points at the potential drawback of the ABC system, which lies in the time and resources consumption associated with developing and managing the implementation process. Everaert et al. (2008) claim that many managers who have tried to implement ABC in their organizations, including healthcare executives, have abandoned the attempt in the face of rising costs and employee irritation.

Observed limitations of the system, which are also present in non-healthcare organizations, are frequently solved by tremendous simplification of the standard ABC system. These tendencies have led to the development of methods such as Time-Driven Activity-Based Costing (Stouthuysen et al., 2009), which is based on the reduction the cost drivers into the time drivers or Simplified Activity-Based Costing (Cao et al., 2006), based on a simplification of the system. For instance Öker (2013) presents implementation of TDABC in one hospital. He presents that TDABC is an easily applicable and effective system for the hospital. The biggest advantage is that the time is considered as a single and exclusive cost driver.

Another problem related to ABC instigation in healthcare, which is obvious from the literature, is defining a suitable cost object. One can discern very different approaches in the literature. For instance, the key cost object outlined in the Udpa study (1996), which is used for cost allocation, is the DRG. The problem is that, in some situations, DRGs as the cost object could lead to distortions when they are broadly based, and include case types that are non-homogenous. Vogl (2013) states that the most controversial and demanding stage of cost calculation at a healthcare establishment is cost apportioning at a patient level. Estimating the patient cost is crucial for reimbursement purposes. Lin et al. (2007) used the individual patient as the cost object, but this again increases the complexity of the system.

We can observe different applications of more advanced costing methods in hospitals worldwide. Numerous studies have looked at utilizing costing methods in hospital organizations. The problem mentioned by many academics is that most ABC implementations are designed as the academic models and have not been fully implemented. One of the most important reasons for this could be limitations of the ABC system, which might be considered as the too complex for easy utilization. Consequently, suitable costing system for a healthcare organization should provide the required information outputs with appropriate complexity level, input data requirements and additional costs.

## 2. Methods and Materials

The objective of the study was to perform a cost analysis of the selected sample of patients delineated under different types of DRG (Psoriasis, Varicose Ulcers, Eczema and other illnesses) in the dermatology department of a Czech regional hospital. The following research questions were defined:

- What is the cost variability of the individual patients within the same DRG?
- What are the differences in the costs of patients receiving inpatient treatment compared to outpatient treatment?
- What are the differences between the costs of patients calculated by the Activity-Based Costing method compared to a traditional costing calculation and the insurance reimbursements received by the hospital?

The hospital selected for cost analysis is a mid-size establishment, with 33 departments of primary care and 13 specialized centers, numbering 2,074 employees and 1,084 beds. The structure of the hospital includes 33 primary care cost centers and 32 support cost centers. The department selected (the Dermatology Department) is a medium-sized primary care department, generating an operating cost of approximately 0.6 mil EUR per year, and boasting personnel of 5 physicians, 13 nurses and 2 other staff. The majority of patients treated at the department consist of those with psoriasis, venous ulcers and eczematous and allergic skin diseases.

Several data sources had been used for the study. Data related to the elementary cost elements was obtained from financial accounting reports. Additional cost data, related to the inter-organizational cost consumption, was obtained from additional management accounting evidence, which is performed additionally to the financial accounting evidence in order to register the consumption of services and related costs between the departments. Both information sources use different software tools.

Another data source has been used for non-financial data. Different specialized data records were used for more accurate cost assignment. For instance, for the distribution of electricity or heating costs we used the specialized evidence kept by the maintenance department.

Non-financial data related to the resource and activity cost drivers was obtained partly from the patient registry and partly from interviews with department employees. Data related to the individual patient and their consumption of activity units was obtained mostly from the patient registry.

Based on interviews with the senior doctor and his assumptions, we expected the following results from the research questions. According to the observed relevant differences between the length of stay and consumption of medications and material between individual patients with the same diagnosis, we expected also relevant differences in cost. Medical practices in recent decades also mostly favor outpatient treatment. The objective of the study was then also to verify this assumption and investigate the “profitability” of inpatient and outpatient treatments in comparison to the costs and revenues of selected patients. We expected also relevant differences between the cost calculated by the ABC method and traditional costing recently used within the department.

This study includes several ethical considerations. The traditional managerial outputs of the ABC system include the use of more accurate cost data for better profitability management and cost optimization. This means that more accurate cost information in comparison with the price of the product (reimbursement) allows the manager to focus on the more profitable products or customers. The ABC approach also allows a better analysis or relations between the consumed costs and performed activities, which could lead to a reduction of ineffective tasks and could identify inappropriate costs. The effective adoption of such actions within healthcare organizations is mostly limited by ethical reasons.

In order to calculate the cost of an individual patient it was first necessary to apply the five consequent steps of the ABC application. The first of these was to process the financial data to make it usable for the ABC method. Direct and indirect costs registered in the department cost center were distinguished. Direct costs, e.g., the medicines prescribed or materials used for individual patients, were allocated directly to the pursued object. Indirect (overhead) costs were allocated through the activities discerned.

The next part of the implementation process was to define the major activities that take place in the department. Consequently, it was necessary to discern those that reflect the significant cost activities of the department. The entire ABC system would be more complex and less clear if too many activities were identified for the department. In this case, the department identified a total of 11 primary activities, making it relatively low in volume compared with other ABC implementations presented, but seemingly sufficient for accurate cost allocation. An overview is shown below:

◾ Outpatient examination
◾ Specialized examination - Dermatoscope
◾ Photo-balneo-therapy
◾ Instrumentation massage - Pneuven
◾ Minor surgical procedures
◾ Patient admission
◾ Hospitalization
◾ Treatment of psoriasis
◾ Treatment of varicose ulcers
◾ Treatment of eczema and other illnesses
◾ Patient release

The primary activities were provided by the Dermatology Department and by selected primary care cost centers, like the surgery department. In order to assign the full cost of individual primary activities, subsequent secondary (infrastructure) activities were identified:

◾ Management and administration
◾ Human resources management
◾ Information systems and technology
◾ Facility management
◾ Medical support services

Support activities represent those provided by the support cost centers.

After identifying the main activities, indirect costs were allocated to them. A different allocation mechanism was utilized for each cost element. Several resource cost drivers were defined, e.g., labor (considered as consumption) by physicians, and quantified by activities, the square meters of the floor space related to activities, etc. All costs were assigned to activities using an Activity Cost Matrix, which displays the relations between the given indirect cost and the activities.

Total cost of primary activities shows Table 1.

**Table 1:** Total cost of primary activities

Activity	Total cost
Outpatient examination	126 743 €
Specialized examination - Dermatoscope	8 788 €
Photo-Balneo-Therapy	43 894 €
Instrumentation massage - Pneuven	11 981 €
Minor surgical procedures	13 073 €
Patient admission	15 470 €
Hospitalization	144 713 €
Treatment of psoriasis	30 756 €
Treatment of varicose ulcers	15 246 €
Treatment of eczema and other illnesses	31 477 €
Patient release	17 610 €

Similarly, the cost of secondary activities was calculated. In this instance, the cost allocation process was much simpler, as only 5 support activities across the entire establishment were identified, and the costs from all the support cost centers were assigned to such support activities.

It was also necessary to determine the amount of costs relating to support activities within the hospital. Another requirement was to quantify the costs of individual centers and divide them into the consequent support activities. It was then necessary to assign a specific portion of these costs to the Dermatology Department. Regarding the activities of management and administration, IS/IT and facility management, the costs of these activities were estimated for the Dermatology Department. The costs were calculated in accordance with the cost of the Dermatology Department in relation to the total cost (the ratio is 0.97%). The costs of human resources management were transposed by the employees of the Dermatology.

Department to the total number of employees - the share is, consequently, 1.02%. The activity labeled “Medical support services includes the various activities performed by the special departments whose outputs are consumed by other primary care departments, i.e. Imaging Methods (X-Ray CT, MRI, etc.), the Department of Clinical Biochemistry, the Department of Medical Microbiology, the Blood Transfusion Department, Nuclear Medicine, the Allergy and Clinical Immunology Laboratory, and the Department of Anatomical Pathology.

A list of these departments is presented in Table 2. Firstly, the portion of the outputs provided by individual departments for the Dermatology Department had to be determined. These data were obtained from the hospital information system, where evidence of the number of examination requests from primary departments is available. In instances when such data were not available, or were registered in an unusable structure, the authors used interviews with the designated persons who estimated the output portions. Due to the total cost of individual support departments being registered in the hospital’s financial statements, after determining the percentages of the departments’ outputs provided for the Dermatology Department, the total costs consumed by the Dermatology Department could be identified (Table 2). These costs (€ 48,479) were then allocated to the Dermatology Department’s activities.

**Table 2:** Allocation of costs of secondary departments (activity)

Support department	Department cost	Dermatology consumption	Dermatology cost
Clinical biochemistry	1 322 089.94 €	1.0808	14 290 €
Microbiology	1 096 812.03 €	2.4602	26 984 €
Radiology I	98 187.59 €	0.6324	621 €
Radiology II	270 148.92 €	0.0147	621 €
Magnetic resonance	298 658.91 €	0.0000	0 €
CT	1 570 914.48 €	0.0337	530 €
Angiography	228 558.30 €	1.0985	2 511 €
Blood transfusion	1 510 087.15 €	0.0411	621 €
Hematology	25 365.66 €	0.4953	126 €
Nuclear medicine	652 142.23 €	0.1550	1 011 €
Pathology	362 620.80 €	0.1808	655 €
Allergy and Immunology	65 433.59 €	0.7802	511 €
**Total**	**7 501 019,59 €**		**48 479 €**

The Table 3 shows the cost of secondary activities and the calculated portion of the cost allocated to the Dermatology Department.

**Table 3:** Total costs of secondary activities

Activity	Total cost	Total cost - Dermatology
Management and administration	1 557 174.11 €	15 097.05 €
Human resources management	65 060.07 €	662.11 €
Information systems and technology	593 228.07 €	5 751.45 €
Facility management	685 315.64 €	6 644.25 €
Medical support services	7 547 424.91 €	47 959.89 €

The next step of the cost calculation process was to calculate activity rates, i.e., activity unit costs. In order to calculate such activity rates it is necessary to perform several steps. Firstly, the cost drivers for each activity had to be determined. We used, for example, the number of examinations, patients, the quantity of patient days and number of hours. Secondly, the output measures for individual activities were identified. The output measure determines the volume of the activity in the period observed. All data were registered in the hospital’s information systems. After gathering the required data, the activity rates had been calculated by the division of total activity cost by the activity output measure estimated. Table 4 shows the result of the performed action in this phase of costing process. The column *total activity cost* shows the cost of activities, including the allocated cost of secondary activities.

**Table 4:** Structure of activities and costs of the dermatology department

Activity	CD	Total activity costs	Output measure	Activity rate
Outpatient examination	number of treatments	150 507.63 €	24 633	6.11 €
Specialized examination - Dermatoscope	number of treatments	9 295.65 €	227	40.95 €
Photo-Balneo-Therapy	number of treatments	47 074.95 €	8047	5.85 €
Instrumentation massage - Pneuven	number of treatments	12 935.20 €	1702	7.60 €
Minor surgical procedures	the number of procedures	13 781.34 €	543	25.38 €
Patient admission	number of patients admitted	16 554.63 €	449	36.87 €
Hospitalization	number of beddays	185 313.00 €	5540	33.45 €
Treatment of psoriasis	number of bed days	32 410.56 €	2352	13.78 €
Treatment of varicose ulcers	number of beddays	16 032.64 €	656	24.44 €
Treatment of eczema and other illnesses	number of beddays	33 143.88 €	2532	13.09 €
Patient release	number of discharged patients	18 640.48 €	452	41.24 €

The final part of cost calculation requires allocating an activity cost to an individual cost object. The ABC system implemented was designed so as to calculate the cost of an individual patient or hospitalization case as the cost object. In order to assign the cost to a cost object, one needs to identify the volumes of individual activity outputs consumed by a specific patient. Final assignation of the cost expended to the specific cost object allows for calculating the total cost per individual patient. Table 5 shows the potential calculation of such patient costs. Direct costs are traced to the cost object directly, while the activity costs are allocated by the volume of the activities’ outputs (output measures) consumed by a cost object.

**Table 5:** Calculation of patient costs

Name of the patient			
Diagnosis	Psoriasis		
Hospitalization			
Direct cost	# of units	Unit cost	Allocated cost
			
			
			
Total direct cost			
Activity name	Output measure	Activity rate	Allocated cost
Outpatient examination	2	6.11 €	12.22 €
Specialized examination - Dermatoscope		40.95 €	0.00 €
Photo-Balneo-Therapy		5.85 €	0.00 €
Instrumentation massage - Pneuven		7.60 €	0.00 €
Minor surgical procedures		25.38 €	0.00 €
Patient admission	1	36.87 €	36.87 €
Hospitalization	6	33.45 €	200.70 €
Treatment of psoriasis	6	13.78 €	82.68 €
Treatment of varicose ulcers		24.44 €	0.00 €
Treatment of eczema and other illnesses		13.09 €	0.00 €
Patient release	1	41.24 €	41.24 €
Total activity cost			373.71 €
Total cost			

## 3. Results

After developing the cost allocation methodology, which adheres to Activity-Based Costing principles and facilitates more accurate cost allocation, a cost analysis of selected patient groups was performed. The patients were divided into three major groups according to the relevant DRG: psoriasis, varicose ulcers and eczema and other illnesses. The objective of the cost analysis was to compare the ABC cost of patients within the defined patient groups and correlate the inpatient and outpatient treatment of the defined DRGs. In order to analyze the cost of patients, a sample of 10 outpatients and 15 inpatients was evaluated.

Initially, analysis was conducted on the patients with psoriasis. 15 hospitalized individuals and 10 outpatients with psoriasis were chosen. Table 6 shows the costs of 15 randomly-selected hospitalized patients with psoriasis. The table firstly displays such costs that were identified based on the ABC method, as described in the *methods* part of the study. It can be seen that the costs range from 219.84 € to 1,370.44 €. Patient 4 generated a very low cost in comparison with the others, the reason being that he was hospitalized for a shorter time and therefore did not consume activities as widely as other patients. The costs are mainly influenced by the length of hospitalization and other treatments received, such as in the case of photo-balneo-therapy for psoriasis. Psoriasis represents a single DRG payment in the amount of 641.38 €. For comparison, the last column presents costs as calculated by KNTB itself. It can be seen that the diameter does not differ significantly, but the actual calculation of the costs to the patient are within +/- 15%. The table below provides data on the minimum, median and maximum values. It is also an important indicator of the level to which funds match up to health insurance payouts. In this case, the average cost per ABC is higher than the average reimbursement from the insurance companies (approximately 73%).

**Table 6:** Costs and payments for patients with psoriasis

Psoriasis - hospitalitation			

	ABC calculated cost	DRG	Traditionally calculated cost
patient 1	1 240.41 €	641.38 €	1 246.11 €
patient 2	968.87 €	641.38 €	1 150.18 €
patient 3	444.14 €	641.38 €	467.35 €
patient 4	219.84 €	641.38 €	189.16 €
patient 5	1 163.67 €	641.38 €	1 145.38 €
patient 6	804.04 €	641.38 €	695.96 €
patient 7	910.21 €	641.38 €	926.69 €
patient 8	656.48 €	641.38 €	668.29 €
patient 9	609.24 €	641.38 €	568.91 €
patient 10	1 116.43 €	641.38 €	1 114.84 €
patient 11	1 163.67 €	641.38 €	1 169.27 €
patient 12	1 069.47 €	641.38 €	1 095.27 €
patient 13	1 370.44 €	641.38 €	1 340.36 €
patient 14	603.39 €	641.38 €	611.89 €
patient 15	904.37 €	641.38 €	881.78 €
average	882.98 €	641.38 €	884.76 €
maximum	1 370.44 €	641.38 €	1 340.36 €
minimum	219.84 €	641.38 €	189.16 €
median	910.21 €	641.38 €	926.69 €
standard deviation	323.35 €	0.00 €	335.02 €
Cost coverage	72.64%		

Table 7 documents 10 patients with psoriasis, their costs and the payouts received from the health insurance companies. When comparing the average cost of treatment and the average insurance payout, it is clear that the costs are lower than said payouts from the insurance companies. The cost of the patient is affected by the number of instances of photo-balneo-therapy and, of course, the number of outpatient visits. The table shows the minimum, maximum and median values. It displays that an average payout from an insurance company covers the cost of treatment by about 178%.

**Table 7:** Costs and payments for ambulatory patients with psoriasis

Psoriasis - outpatient		

	ABC calculated cost	Reimbursement
patient 1	125.72 €	318.44 €
patient 2	496.76 €	1 100.47 €
patient 3	371.04 €	898.95 €
patient 4	18.35 €	14.76 €
patient 5	64.56 €	73.20 €
patient 6	122.99 €	160.69 €
patient 7	129.11 €	44.62 €
patient 8	275.09 €	689.49 €
patient 9	30.58 €	20.76 €
patient 10	356.75 €	217.60 €
Average	199.10 €	353.90 €
maximum	496.76 €	1 100.47 €
Minimum	18.35 €	14.76 €
Median	127.42 €	189.15 €
Standard deviation	125.72 €	318.44 €
Cost coverage	177.75%	

A second analysis was carried out for patients suffering from varicose ulcers. Similarly, as for psoriasis patients, 15 hospitalized patients and 10 outpatients with varicose ulcers were chosen for analysis. Table 8 shows the costs of 15 randomly selected hospitalized patients with varicose ulcers. As can be seen, the cost variability between the individual patients is slightly higher than for psoriasis. Major differences could also be identified between the ABC calculated costs and those worked out via traditional methodology. The average cost calculated by ABC is 22% higher than the figure calculated in the traditional manner. As can be seen, reimbursement covers just 61% of the average cost of the DRG.

**Table 8:** Costs and reimbursements for hospitalized patients with varicose ulcers

Varicose Ulcers - hospitalization			

	ABC cost	DRG	Traditional cost
patient 1	774.47 €	641.38 €	638.29 €
patient 2	1 152.34 €	641.38 €	752.98 €
patient 3	1 144.76 €	641.38 €	740.80 €
patient 4	1 464.71 €	641.38 €	1 223.45 €
patient 5	1 137.17 €	527.60 €	672.22 €
patient 6	643.46 €	641.38 €	512.69 €
patient 7	396.60 €	641.38 €	209.71 €
patient 8	1 048.90 €	641.38 €	593.71 €
patient 9	1 464.71 €	641.38 €	1 265.42 €
patient 10	605.52 €	641.38 €	390.33 €
patient 11	605.52 €	641.38 €	1 948.22 €
patient 12	1 623.29 €	641.38 €	1 153.02 €
patient 13	1 071.66 €	641.38 €	586.44 €
patient 14	1 493.33 €	641.38 €	1 212.18 €
patient 15	847.57 €	641.38 €	724.62 €
Average	1 031.60 €	633.80 €	841.60 €
Maximum	1 623.29 €	641.38 €	1 948.22 €
Minimum	396.60 €	527.60 €	209.71 €
Median	1 071.66 €	641.38 €	724.62 €
standard deviation	377.57 €	29,38 €	441.08 €
Cost coverage	61.44%		

Table 9 shows a similar analysis of outpatient treatment, but here the cost of patients varies dramatically from 12.23 € to 467.52 €. Reimbursement is not made by a fixed rate per DRG, but is calculated as the sum of the tasks performed. Average reimbursement is 17% higher than the average ABC costs.

**Table 9:** Costs and reimbursements for outpatients with varicose ulcers

Varicose Ulcers - outpatients		

	ABC costs	DRG
patient 1	18.35 €	20.76 €
patient 2	108.16 €	106.11 €
patient 3	30.58 €	46.29 €
patient 4	116.21 €	197.75 €
patient 5	12.23 €	15.31 €
patient 6	347.58 €	451.82 €
patient 7	467.52 €	238.40 €
patient 8	82.00 €	347.05 €
patient 9	163.99 €	83.20 €
patient 10	37.94 €	25.38 €
average	138.46 €	153.21 €
maximum	467.52 €	451.82 €
minimum	12.23 €	15.31 €
median	95.08 €	94.65 €
standard deviation	152.52 €	151.82 €
Cost coverage	110.65%	

A final analysis was made for eczema and other illnesses. Similarly, 15 hospitalized individuals and 10 outpatients were chosen for analysis. Table 10 shows the costs of 15 randomly-selected hospitalized patients with eczema and other illnesses.

**Table 10:** Costs and reimbursements for hospitalized patients with eczema and other illnesses

Eczema and otherillnesses - hospitalization			

	ABC calculated costs	DRG	Traditionally calculated costs
patient 1	456.68 €	466.51 €	517.42 €
patient 2	503.24 €	466.51 €	599.67 €
patient 3	270.46 €	466.51 €	246.22 €
patient 4	786.84 €	466.51 €	784.36 €
patient 5	223.90 €	334.15 €	207.75 €
patient 6	188.23 €	334.15 €	251.09 €
patient 7	270.46 €	334.15 €	254.91 €
patient 8	596.35 €	466.51 €	652.18 €
patient 9	363.57 €	334.15 €	435.93 €
patient 10	410.13 €	334.15 €	444.69 €
patient 11	736.02 €	334.15 €	651.89 €
patient 12	363.57 €	334.15 €	405.64 €
patient 13	217.79 €	334.15 €	289.20 €
patient 14	782.58 €	334.15 €	971.89 €
patient 15	782.58 €	334.15 €	947.89 €
Average	463.49 €	378.27 €	510.72 €
maximum	786.84 €	466.51 €	971.89 €
Minimum	188.23 €	334.15 €	207.75 €
Median	410.13 €	334.15 €	444.69 €
Standard deviation	222.42 €	64.59 €	252.31 €
Cost coverage	81.61%		

Table 11 shows a similar analysis for outpatient treatment. In this case, the sample group was extended to 30 patients.

**Table 11:** Costs and reimbursements for outpatients with eczema and other illnesses

Eczema and otherillnesses - outpatients		

	ABC calculatedcosts	DRG
patient 1	18.35 €	35.09 €
patient 2	82.36 €	152.07 €
patient 3	30.58 €	24.40 €
patient 4	6.12 €	7.35 €
patient 5	12.23 €	14.69 €
patient 6	47.07 €	18.11 €
patient 7	6.12 €	7.35 €
patient 8	12.23 €	14.69 €
…		
patient 30	18.35 €	15.64 €
average	27.38 €	21.88 €
maximum	94.13 €	152.07 €
minimum	6.12 €	4.58 €
median	21.41 €	14.60 €
standard deviation	23.06 €	26.64 €
Cost coverage	79.93%	

## 4. Discussion

The performed cost analysis led to several important results and implications for management. Firstly, the study confirmed the expected result, i.e., that the cost of individual patients within the selected diagnoses is diverse. Cost diversity is caused by the differences in the activity outputs consumed by the individual patients. Another important implication arises through comparing the costs calculated by ABC and the reimbursements received. Such reimbursements for hospitalization treatment are, under present conditions in the Czech Republic, arrived at through a fixed payment for the relevant DRG. Cost analysis showed that the average costs of these patients are only partially covered by such reimbursements. Nevertheless, reimbursements paid out for outpatient treatment are worked out according to the direct payment per task defined. In this case, average reimbursement payouts are higher than the costs calculated by ABC. This means that the yield from outpatient activities goes towards covering the costs of hospitalization. The department could then improve cost efficiency through changing the type of treatment offered from hospitalization to outpatient care. This could lead to reduced expenditures and increased reimbursements from the insurance companies.

Additionally, interesting results were found through comparing the ABC cost calculated via the newly designed ABC system and traditional cost calculations, as based on a fixed daily rate per patient day and the individual evaluation of the several direct activities chosen. The group of patients with psoriasis showed little difference between the cost calculated via ABC and the traditional approach. This is due to the relatively standardized manner of treatment given. However, for varicose ulcer patients, the variations between the ABC and traditionally-calculated costs could prove important in terms of diagnoses, where sizeable differences exist between patients. This means that the way in which individual patients are treated may well vary. In the case of diagnoses where treatment is performed in a more standardized way and hence the difference in the treatment received by individual patients is not important, both costing methods arrive at similar results.

Using the ABC system to calculate the cost of patients and consequent cost analysis brings with it several major benefits. The ABC method could be considered as more accurate and relevant than the traditional costing method. Greater accuracy results from the better detailed structure of the cost pools - the activities and wider variety in the forms of cost drivers. The outputs of ABC costing could potentially create an abundance of additional information for managers. For instance, they would be able to analyze the total cost of activities, analyze the output of the activities, measure the level of utilization of individual activities, and measure the impact on the cost of a patient. Reaching a sufficient level of utilization could prove important in instances of costly attempts at treatment using, e.g., highly expensive equipment.

Despite the increased accuracy of the ABC system developed, many reasons still prevail as to why the costing method utilized herein might not be considered as one of absolute accuracy. Allotting an overhead cost is always based on a specific proportion of arbitrary allocation. According to the nature of the overhead cost, we do not have any real way to verify the accuracy of the cost allocation process. Based on theory, we expect higher accuracy from the ABC system, but a poorly-designed system could in some cases lead to more inaccurate cost assignments than with the traditional costing approach. A major limitation of the model presented is in allocating the costs of medical support services. Despite the fact that we conducted a detailed analysis of costs related to the Dermatology Department, further allocation to the individual patient was arrived at via an arbitrary principle. Inaccurate allocation of the medical support services could distort the final cost calculation. However, this issue might be solved relatively easily by transposing such medical support services into a standard activity structure. Nevertheless, this would add greatly to the complexity of the system as it results in dramatically increasing the number of activities at the very least. Having said this, the cost of medical support services allocated to the Dermatology Department was only 8% of the total cost.

Another important limitation of the presented study is the time and resource requirements for the data processing. It is an accepted fact that using the ABC method is much more demanding in terms of data than a traditional cost evaluation. Applying the costing system at the Dermatology Department took one month to implement. The present study includes the ABC model, which is relatively simple in comparison with other ABC healthcare models in number of activities and used cost drivers. The most time-consuming step was allotting the cost registered in the accounting system to the defined structure of activities. It was also necessary to acquire additional data from the various departments related to accurate overhead cost allocation, such as the proportion of energy consumption related to individual departments, etc. Acquiring other additional data from the system of patient evidence was also necessary. However, once such an ABC system is implemented, and the relations between the costs, activities and cost objects are established in the costing system, its future operation is more simple. Furthermore, due to the high quality of data evidenced on the individual patients, it could be automated relatively easily.

A correctly implemented ABC system offers a good alternative to traditional costing, allowing for greater accuracy that would in turn provide a more precise cost analysis. Nevertheless, it would be necessary to avoid the excessive structure of the ABC model, which could lead to data overload.

As mentioned in the *methods* sections, the managerial implications of the performed study is more limited in comparison with business organizations. A hospital organization could not so easily manage the portfolio of the customers/patients. Also, the simple methods of cost reduction based on the study of relationships between the consumed costs and activities and identification of inappropriate costs could be not so easily performed like in the case of business organizations due to the crucial focus on treatment failures or errors.

## 5. Conclusion

The most important benefit of the system herein described is the ability to calculate more accurately the cost of an individual patient. The method highlights differences between the costs of an individual patient under the same diagnosis and their differing demands on hospital activities. Traditional costing systems mostly determine average costs, failing to note any differences between individual patients. If one lacks information on such cost differences, one is unable to analyze the causes of those differences. Calculating a more accurate cost of an individual patient may also result in “profitability” or verifying the cost coverage of patients or the diagnosis types presented in the study. Comparing the calculated cost and revenue (reimbursement) for an individual patient enables the user to discern types of patient with a diagnosis that could financially benefit the hospital, as well as those for which the expenditure required would not be covered. As can be seen, many diagnoses result in a low amount of reimbursement, which has a crucial impact on the profitability of a healthcare organization. Identifying the DRGs of patient types of low profit could lead to appropriate managerial decisions.
